# Characterization of a cyanobacterial rep protein with broad-host range and its utilization for expression vectors

**DOI:** 10.3389/fmicb.2023.1111979

**Published:** 2023-03-23

**Authors:** Yutaka Sakamaki, Kaisei Maeda, Kaori Nimura-Matsune, Taku Chibazakura, Satoru Watanabe

**Affiliations:** ^1^Department of Bioscience, Tokyo University of Agriculture, Tokyo, Japan; ^2^Laboratory for Chemistry and Life Science, Institute of Innovative Research, Tokyo Institute of Technology, Yokohama, Japan

**Keywords:** cyanobacteria, expression vector, broad host range, library screening, next-generation sequencing

## Abstract

Owing to their photosynthetic capabilities, cyanobacteria are regarded as ecologically friendly hosts for production of biomaterials. However, compared to other bacteria, tools for genetic engineering, especially expression vector systems, are limited. In this study, we characterized a Rep protein, exhibiting replication activity in multiple cyanobacteria and established an expression vector using this protein. Our comprehensive screening using a genomic library of *Synechocystis* sp. PCC 6803 revealed that a certain region encoding a Rep-related protein (here named Cyanobacterial Rep protein A2: CyRepA2) exhibits high autonomous replication activity in a heterologous host cyanobacterium, *Synechococcus elongatus* PCC 7942. A reporter assay using GFP showed that the expression vector pYS carrying CyRepA2 can be maintained in not only *S.* 6803 and *S.* 7942, but also *Synechococcus* sp. PCC 7002 and *Anabaena* sp. PCC 7120. In *S.* 7942, GFP expression in the pYS-based system was tightly regulated by IPTG, achieving 10-fold higher levels than in the chromosome-based system. Furthermore, pYS could be used together with the conventional vector pEX, which was constructed from an endogenous plasmid in *S.* 7942. The combination of pYS with other vectors is useful for genetic engineering, such as modifying metabolic pathways, and is expected to improve the performance of cyanobacteria as bioproduction chassis.

## 1. Introduction

Cyanobacteria are the predominant phototrophs in ocean and freshwater ecosystems, and are among the most widespread phylogenetic clades. Cyanobacteria have oxygen-producing photosynthetic capabilities, meaning that they can produce biomass using solar energy and CO_2_, and have recently gained attention for their potential as green cell factories for CO_2_-neutral biosynthesis of various products (Knoot et al., 2018; Farrokh et al., 2019). Recently, three cyanobacterial model strains *Synechocystis* sp. PCC 6803 (*S*. 6803), *Synechococcus elongatus* PCC 7942 (*S*. 7942), and *Synechococcus* sp. PCC 7002 (*S*. 7002) have been used in synthetic biology studies for the biosynthesis of multiple products including biofuels (Lan and Liao, 2011; Liu et al., 2011), antioxidants (Shimada et al., 2020), flavors/fragrances (Formighieri and Melis, 2015), and pharmaceuticals (Choi et al., 2016). The multicellular filamentous cyanobacterium, *Anabaena* sp. PCC 7120 (*A*. 7120), which performs fixation of both nitrogen and carbon, is particularly suitable for the production of nitrogenous substances, and has recently been studied for ammonia production (Higo et al., 2016).

In several model cyanobacteria, *S*. 7942 (Taton et al., 2020), *S*. 6803 (Englund et al., 2015; Chin et al., 2018), and *S*. 7002 (Wang et al., 2019), integration of genetic constructs into chromosomal neutral sites has been used for exogenous gene expression. Due to their polyploidy, chromosomal expression systems in these cyanobacteria are expected to be more effective than those in monoploid organisms, although genetic manipulation is limited to a few cyanobacteria that have natural competence and chromosomal recombination abilities. In addition, genetic engineering relying on integration at neutral sites is time-consuming when performed sequentially, because all chromosomes must be segregated to stably retain the genetically modified constructs in polyploid cyanobacteria.

Self-replicating plasmids are maintained autonomously using replication initiation factors (e.g., Rep proteins) encoded in the plasmids, and harnessing replication machinery of the host. The autonomously replicating sequences consist of an initiation factor and its binding sequence, which serves as the replication origin; however, the binding sequence can also function alone if the host’s replication initiation factor functions in *trans*. Most plasmid replicons in *Escherichia coli* cannot be used directly in cyanobacteria, and there is little information on autonomously replicating sequences and available plasmid vectors in cyanobacteria. The broad-host-range plasmid RSF1010, a member of the IncQ plasmid, stably replicates in a wide variety of Gram-negative (Meyer, 2009) and Gram-positive bacteria (Trieu-Cuot et al., 1987), including *E. coli* and several strains of cyanobacteria (Cassier-Chauvat et al., 2021). The RSF1010 vectors are maintained in *S*. 6803 cells but are not suitable for overexpressing genes because their copy number is comparable to that of chromosomes (Jin et al., 2018). Other vectors utilizing cyanobacterial endogenous plasmids have also been developed, including pUH24/pANS (and the derivative vector pUC303) in *S*. 7942 (Kuhlemeier et al., 1983; Van der Plas et al., 1992), pCA2.4, pCB2.4 (pSOMA series; Opel et al., 2022), and pCC5.2 (pSCB; Jin et al., 2018) in *S*. 6803, pAQ (pAQ1-EX1) in *S*. 7002 (Miyasaka et al., 1998), and pDU1 (pRL series) in *Nostoc* sp. PCC 7524 (Wolk et al., 1984). However, these vectors have not been tested for their host range, except for pSOMA, which has been recently demonstrated to be maintained in two cyanobacteria *S*. 6803 and *A*. 7120 (Opel et al., 2022). Further developments in cyanobacterial genetic engineering will require autonomously replicating regions that function in a wide range of species.

In this study, toward the development of broad host-range vectors, we screened autonomously replicating regions and found that a region containing Rep-related protein ORF B (here named Cyanobacterial Rep protein A2: CyRepA2) encoded in the plasmid pCC5.2, derived from *S*. 6803, has replication activity in *S*. 7942. Using this region, we constructed an expression vector, pYS, and tested its expression efficiency, host range, and compatibility with other plasmids. Our results not only contribute to elucidating Rep proteins and their regulatory mechanisms, which are poorly known in cyanobacteria, but also improve the performance of cyanobacteria as bioproduction chassis.

## 2. Materials and methods

### 2.1. Cyanobacteria strains and growth condition

The freshwater cyanobacteria *Synechococcus elongatus* PCC 7942 (our laboratory strain *S*. 7942 TUA, which lacks the endogenous small plasmid pHU24/pANS; Watanabe et al., 2012), and *Synechocystis* sp. PCC 6803 (sub-strain *S*. 6803 GT-I; Kanesaki et al., 2012) were used in this study. Both strains were cultured in modified BG-11 medium, which contained double the usual amount of sodium nitrate (final concentration 35.3 mM) and 20 mM HEPES-KOH (pH 8.2), while *Anabaena* sp. PCC 7120 was maintained in standard BG11 (Castenholz, 1988). The marine cyanobacterium *Synechococcus* sp. PCC 7002 was cultured in modified A2 medium containing one-third the usual amount of sodium nitrate (final concentration 17.3 mM; Hasunuma et al., 2019). Cultures were grown photoautotrophically at 30°C, using continuous illumination (50 μmol photons m^−2^ s^−1^) and with 2% CO_2_ (v/v) bubbling. When appropriate, spectinomycin (*S*. 7942: final concentration 40 μg/mL, *A*. 7120: 10 μg/mL) or chloramphenicol (*S*. 7942 and *S*. 6803: 7.5 μg/mL, *S*. 7002: 20 μg/mL) were added to media.

### 2.2. Autonomous replication sequencing

Each DNA fragment was amplified using PrimeSTAR DNA polymerase (TaKaRa, Shiga, Japan) and subcloned into vectors using T4 DNA ligase (TaKaRa) or an In-Fusion HD Cloning Kit (TaKaRa). For the construction of a *S*. 6803 genomic library, the ColE1 origin and the chloramphenicol acetyltransferase gene (*cat*) were PCR-amplified from pUC4K (Amersham, Chicago, IL, United States) and pUC303 (Kuhlemeier et al., 1983) plasmids using the respective primer sets (F1/R2, F3/R4, Supplementary Table S1) and recombined by PCR (primers: F3/R2). After digestion by *Bam*HI and dephosphorylation treatment using shrimp alkaline phosphatase (TaKaRa), the DNA fragment was ligated with 1.5–2.5 kbp fragments of the *Sau*3AI-digested *S*. 6803 genomic DNA. After transformation of *E. coli,* we obtained 2.5 × 10^4^ clones (Library A). To determine the regions responsible for replication in heterologous cyanobacteria, we transformed *S*. 7942 cells with the *S*. 6803 genomic library to yield 335 colonies, which were then pooled (Library B). To obtain plasmids that replicated independently of chromosomal integration, the DNA extracted from library B was introduced into *E. coli* again, and 1.4 × 10^4^ transformants were obtained (Library C). Genomic libraries (Libraries A–C) were subjected to comprehensive sequencing analysis.

The sequencing library was constructed using the NEB Next Ultra DNA Library Prep Kit (NEB, Ipswich, MA, United States) and analyzed using a MiSeq sequencer with a paired-end 150 bp sequence read run with the MiSeq reagent kit v3 (Illumina, San Diego, CA, United States). The reads were trimmed using the CLC Genomics Workbench ver. 9.5.4 (QIAGEN, Venlo, Netherlands) with the following parameters: Phred quality score > 30; ambiguous nucleotides allowed:1; automatic read-through adaptor trimming: yes; removing the terminal 15 nucleotides from the 5′ end and five nucleotides from the 3′end; and removing truncated reads of less than 30 nucleotides in length. To identify the regions included in the library, trimmed reads were mapped to the *S.* 6803 genome (accession numbers, chromosome: AP012276, pSYSM: AP004310, pSYSX: AP006585, pSYSA: AP004311, pSYSG: AP004312, pCA2.4: CP003270, pCB2.4: CP003271, and pCC5.2: CP003272) using the CLC Genomics Workbench ver. 20.0.1 (QIAGEN) with the following parameters: match score, 1; mismatch cost, 2; indel cost, 3; length fraction, 0.8; similarity fraction, 0.9; and non-specific match handling, ignored. The number of raw read pairs per sample and the ratio of reads mapped on the reference sequences are shown in Supplementary Table S2; Supplementary Figure S1A. Original sequence reads were deposited in the DRA/SRA database with the following accession numbers (Library A: DRR285589, Library B: DRR285594, and Library C: 285596). The accession number for the BioProject was PRJDB11466.

### 2.3. Phylogenetic analysis

**Figure 1:**
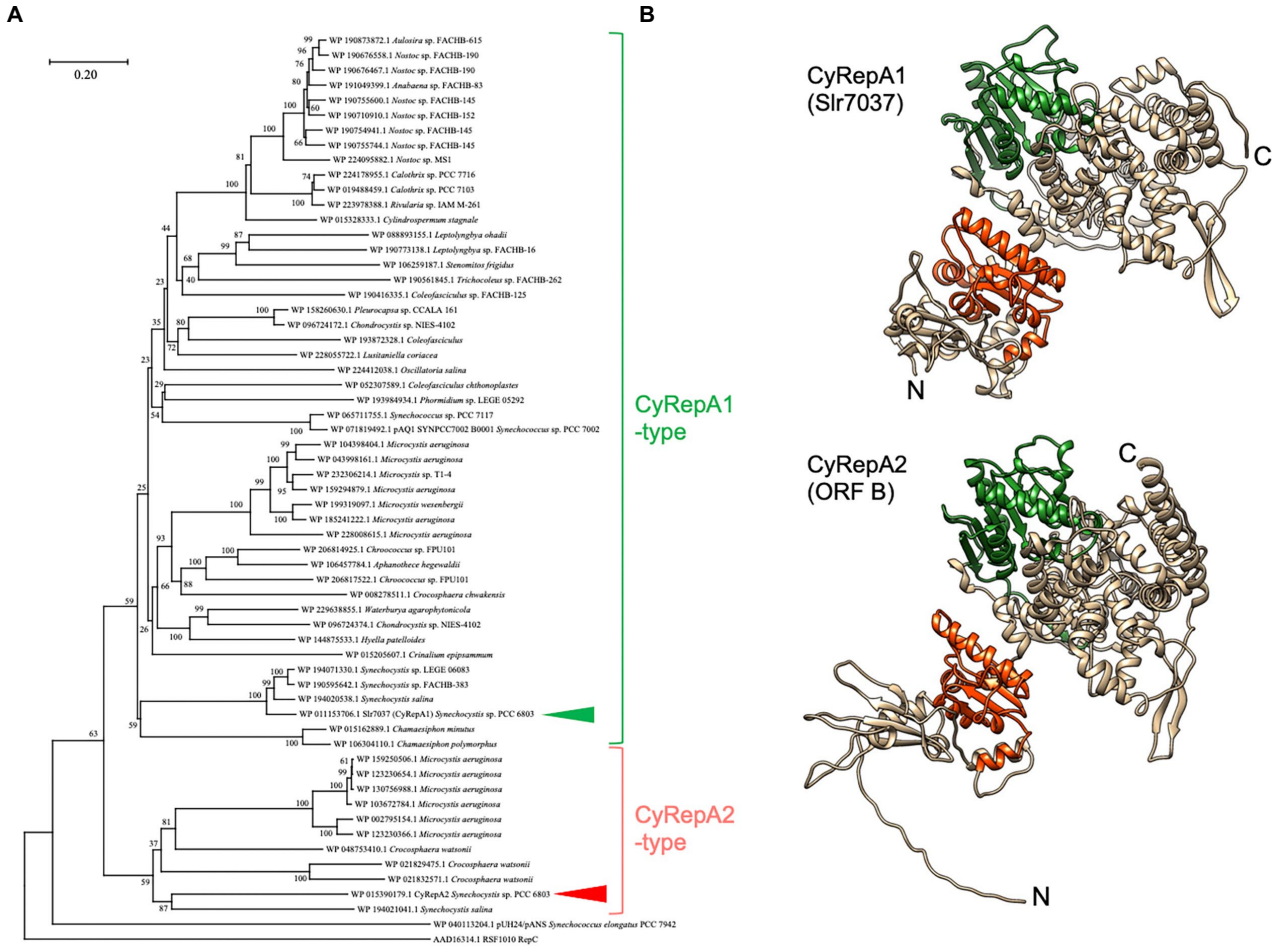
Phylogenetic and structural analysis of Cyanobacterial Rep protein A (CyRepA1: Slr7037; CyRepA2: ORF B). **(A)** Phylogenetic tree of CyRepA. CyRepA2 (ORF B) and its 58 homologs were used for phylogenetic analysis along with pUH24/pANS Rep and RSF1010 RepC used as outgroups. Proteins used for analysis were shown by their accession numbers in database with organism names. The tree is drawn to scale, with branch lengths in the same units as those of the evolutionary distances used to infer the phylogenetic tree. CyRepA1 (Slr7037) and CyRepA2 are indicated by arrow heads. **(B)** 3D structures of CyRepA1 and CyRepA2 predicted using AlphaFold2. The 3D structures are color-coded as follows based on the results of domain predictions by SMART and Pfam. DUF3854 domain (orange), DEXDc domain (green).

The amino acid sequence of ORF B (accession number: WP_015390179.1) was obtained from the database, and its homologs were retrieved by an NCBI-BLAST search for the top 100 most similar sequences. Among these, we excluded overlapping sequences and obtained 58 homologous sequences. Phylogenetic analysis was performed on these homologs, along with pUH24/pANS Rep in *S*. 7942 and RSF1010 RepC, using ClustalW within MEGA11, with default parameters (Tamura et al., 2021). Evolutionary history was inferred using the neighbor-joining method (Saitou and Nei, 1987). The optimal tree is shown in Figure 1A. The percentage of replicate trees in which the associated taxa clustered together in the bootstrap test (1,000 replicates) is shown next to the branches (Felsenstein, 1985). The tree was drawn to scale, with branch lengths in the same units as those of the evolutionary distances used to infer the phylogenetic tree. The evolutionary distances were computed using the Poisson correction method (Zuckerkandl and Pauling, 1965) and were expressed as number of amino acid substitutions per site. This analysis involved 61 amino acid sequences (ORF B, pUH24/pANS Rep, RSF1010 RepC, and 58 homologs). All ambiguous positions were removed for each sequence pair (pairwise deletion). There were 1,655 positions in the final dataset. Evolutionary analyses were conducted using MEGA11 (Tamura et al., 2021).

### 2.4. Prediction of 3D structure of CyRepAs

AlphaFold2 was used for 3D structure prediction (AlQuraishi, 2019; Cramer, 2021). The predicted 3D structure of Slr7037 (CyRepA1) was obtained from the AlphaFold Protein Structure Database. ORF B (CyRepA2) was predicted by using ColabFold and the model with the best score was adopted (Mirdita et al., 2022). The predicted 3D structures were colored to domains using UCSF Chimera (University of California San Francisco, San Francisco, CA, United States; Pettersen et al., 2004). Domain predictions were performed using SMART (Letunic et al., 2021) and Pfam software (Mistry et al., 2021). Predicted 3D structure data of CyRepA1 and CyRepA2 are available in Supplementary Data 1, 2.

### 2.5. Plasmid and strain construction

To construct the expression vector pYS1C-GFP, we used a plasmid isolated from *E. coli* transformant pools of Library B (Supplementary Table S3). DNA fragments containing the *lacI* gene, the *trc* promoter, the *gfp ^mut2^* gene (Cormack et al., 1996), and the B0011 *lux* operon terminator were PCR-amplified using appropriate primer sets (F5/R6, F7/R8, F9/R10, F11/R12, and F13/R14) and introduced into the plasmid obtained from library screening and containing ColE1, *cat*, and a part of pCC5.2 (1,174–4,674 nt) by In-Fusion cloning (Supplementary Figure S2; Supplementary Data 3).

To enable strict control of gene expression in *S*. 7002, the promoter region of pYS1C-GFP was replaced with the *clac143* promoter, which was optimized for regulated expression in *S*. 7002. Using primers F15/R16 flanking the sequence of the *clac143* promoter, DNA fragments were PCR-amplified from pYS1C-GFP and circularized using In-Fusion cloning. The resulting plasmid was designated pYS4C-GFP (Supplementary Figure S2; Supplementary Data 4). To replace the *cat* gene in pYS1C-GFP and pYS4C-GFP with the spectinomycin-resistance (*Sp^R^*) gene, the plasmid regions and *Sp^R^* gene were PCR-amplified using appropriate primer sets (F17/R18, F19/R20) and combined using In-Fusion cloning. The resulting plasmids were designated pYS1S-GFP and pYS4S-GFP.

For the construction of the integration plasmid pNSG, a DNA fragment of the *gfp ^mut2^* gene was PCR-amplified with primers F21/R22 and cloned into the *Sal*I and *Hind*III sites of the integration vector pNSE1 (Kato et al., 2008), containing a region homologous to a neutral site in *S*. 7942. To construct a plasmid harboring the pUC303-based replication system, the region containing *repA* and *repB* of pUC303, the p15A origin, a DNA fragment containing *Sp^R^* gene, and the *mScarlet* gene under the control of the *cpcB* promoter were PCR-amplified using appropriate primer sets (F23/R24, F25/R26, F27/R28) and combined using In-Fusion cloning to form a plasmid designated pEX2S-mScarlet.

To transform the cyanobacteria *S*. 7942, *S*. 6803, and *S*. 7002, cells were grown as described above until they reached an OD_750_ of 0.7–1.2 and then collected by centrifugation at 3,000 × *g* for 10 min at 25°C. After resuspension in BG11 to 10-fold concentration, plasmid DNA was added and incubated at 25°C overnight under light-shielded and rotating conditions. The samples were then irradiated with light for 1 h and spread on drug-containing plates. In the case of *A*. 7120 transformation, the plasmids pYS1S-GFP or pYS4S-GFP were transformed into *E. coli* HB101 carrying the plasmid pRL623 (Elhai et al., 1997) and were transferred to *A*. 7120 cells with the help of the conjugative plasmid RP4, according to the triparental mating method (Thiel and Wolk, 1987; Elhai and Wolk, 1988) with minor modifications. *Escherichia coli* cultures carrying the donor plasmid were mixed with the *E. coli* culture carrying RP4 and incubated for 1 h. The *A*. 7120 culture prepared as described above was added to the *E. coli* mixture and incubated overnight on a BG11 plate containing 5% LB medium. The mixture was harvested by adding BG11 medium to the plate and spread on plates containing spectinomycin.

The structures of the pYS plasmids introduced into cyanobacteria were confirmed by the following procedure: DNA extracted from cyanobacteria harboring pYS plasmids was used to transform *E. coli* JM109. After selection in LB medium containing chloramphenicol or spectinomycin, plasmid DNA was extracted by using the FavorPrep™ Plasmid DNA Extraction Mini Kit (FAVORGEN, Ping Tung, Taiwan). Plasmid DNA samples were digested with *Nde*I and *Pst*I for pYS1C-GFP and pYS4C-GFP, and *Sal*I and *Nhe*I for pEX2S-mScarlet. Each digested DNA sample (80 ng) was subjected to 1% agarose gel electrophoresis.

**Figure 2:**
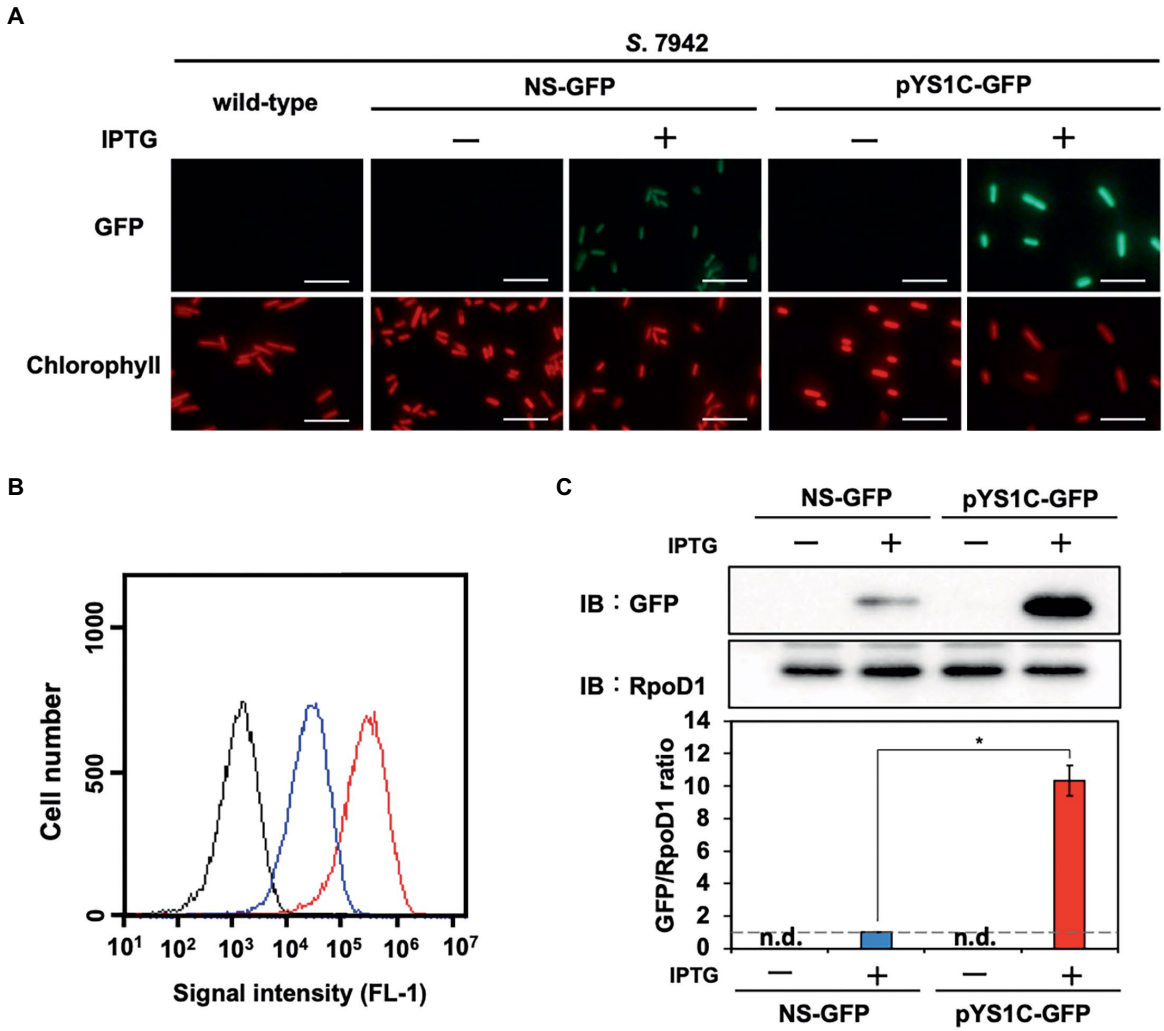
Comparison of expression performance between chromosome and pYS1 plasmid by the GFP reporter system in *S*. 7942. The GFP expression levels in the pYS1C-GFP transformant were compared to *Synechococcus elongatus* PCC 7942 (*S*. 7942) wild-type and NS-GFP strains expressing GFP at the chromosomal neutral site. GFP expression was induced by the addition of 1 mM IPTG (final concentration). **(A)** Fluorescence microscopy images. The GFP and chlorophyll fluorescence images are shown. White bar, 10 μm. **(B)** FACS analysis of the GFP fluorescence. Signal intensity of FL-1 indicating GFP fluorescence in the wild-type (black), NS-GFP (blue), and pYS1-GFP (red) strains are shown. **(C)** Western blot analysis. The protein extracts obtained from wild-type, NS-GFP, and pYS1C-GFP strains were subjected to SDS-PAGE and analyzed by western blotting using antibodies against GFP. RpoD1 was used as an internal control. The signal intensities of GFP were normalized to those of RpoD1, and the ratio the GFP signal to NS-GFP was set to 1. Bars represent the mean ± SEM (*n* = 3; *p* < 0.05).

**Figure 3:**
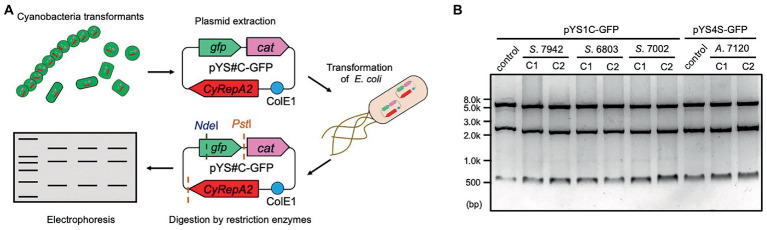
Plasmid structure of pYS in cyanobacteria cells. **(A)** Scheme of the analysis of plasmid structure. To determine whether plasmids are maintained in cyanobacterial cells in a circular structure, DNA was extracted from cyanobacteria transformants (*S*. 7942, *S*. 6803, *S*. 7002, and *A*. 7120) carrying pYS1C-GFP or pYS4S-GFP and introduced into *E. coli*. After the plasmid was extracted from the *E. coli* cells, plasmids were digested with restriction enzymes, and compared to the plasmids before the transformation of cyanobacteria. **(B)** Electrophoresis image of plasmids digested by the restriction enzymes. The plasmids before transformation were used as controls. The results of two independent clones are shown as C1 and C2.

### 2.6. SDS-PAGE and western blotting analysis

The protein extracts from cyanobacteria cells were prepared according to the method described by Nimura et al. (2001) and were separated by SDS-PAGE on 12% polyacrylamide gels at 150 V for 90–100 min in electrophoresis buffer (25 mM Tris, 192 mM glycine, and 0.1% SDS). The gels were blotted onto 0.2 μm PVDF membranes (Bio-Rad, Hercules, CA, United States) at 2.5 A for 10 min using a Trans-Blot Turbo Transfer System (Bio-Rad). Membranes were blocked for 30 min with 5% skimmed milk in TBS-T [50 mM Tris–HCl (pH 7.5), 150 mM NaCl, 0.05% Tween 20], washed, and soaked in TBS-t for 2 h at room temperature with anti-GFP (MBL, Tokyo, Japan), anti-RpoD1 rabbit antiserum (Seki et al., 2007), or anti-RbcL (Agrisera, Vännäs, Sweden) as primary antibodies. After washing, the membranes were soaked in TBS-t for 30 min at room temperature with HRP-conjugated anti-mouse (GE Healthcare, Chicago, IL, United States) or anti-rabbit antibody (GE Healthcare) as secondary antibodies. Chemiluminescence was detected with the LumiGLO Chemiluminescent Substrate KPL using ChemiDoc XRS Plus (Bio-Rad).

### 2.7. Flow cytometry

Cells were analyzed by GFP fluorescence-activated cell sorting (FACS) using a BD Accuri™ C6 Flow cytometer (BD Biosciences, San Jose, CA, United States) and BD CFlow software (BD Biosciences), as described by Watanabe et al. (2012). Cyanobacterial cells showing chlorophyll fluorescence were sorted using the FL3 channel and GFP fluorescence was measured using the FL1 channel.

### 2.8. Microscopy

Fluorescent images were obtained using BX53 microscope (OLYMPUS, Tokyo, Japan) at ×40 or 100 magnification with a DP71 digital camera (OLYMPUS) and DP Controller software ver. 3. 3. 1. 292 (OLYMPUS).

## 3. Results

### 3.1. Library screening of sequences responsible for autonomous replication activity in *Synechococcus elongatus* PCC 7942

To gain new insights into autonomously replicating regions in cyanobacteria, we established a new method, Autonomous Replication sequencing (AR-seq), which combines library screening and sequencing. As a source organism for screening, we selected *S*. 6803 (library A), which contains seven plasmids in addition to the chromosome, and at least seven replicons that can coexist. *S*. 7942 was selected as the host cyanobacteria for transformation with the plasmid-based *S*. 6803 genomic library for three reasons: avoiding chromosome-library recombination, having extremely high transforming ability, and containing only one large plasmid pANL. A genomic library of 2.5 × 10^4^ clones (library A) was constructed by ligating 1.5–2.5 kbp fragments of *S*. 6803 genomic DNA with the ColE1 origin and *cat* gene, and this was introduced into *S*. 7942. As a result, more than 300 transformants were obtained. DNA extracted from *S*. 7942 transformants was pooled as library B, which contained the autonomously replicating sequences in addition to *S*. 7942 genomic DNA. To isolate plasmids that replicated independently of chromosomal integration in *S*. 7942 cells, library B was again introduced into *E. coli*, and only the plasmid DNA was pooled as library C. At this stage, 14 *E. coli* colonies were picked from the transformants of library B, and the inserts were sequenced by Sanger sequencing. The results showed that all colonies contained the region surrounding ORF B of pCC5.2, a plasmid found in *S*. 6803 (Supplementary Table S3).

Next, a comprehensive sequencing analysis was performed to reveal the genomic regions in the libraries. The results showed that pCC5.2 occupied a large proportion of the library in spite of its relatively short DNA length in the *S*. 6803 genomic library A (Supplementary Figure S1A; Supplementary Table S2), suggesting a high copy number of pCC5.2 in *S*. 6803 cells, as reported previously (Jin et al., 2018). The fact that only a few sites were recognized by *Sau*3AI in pCA2.4 and pCB2.3 may have contributed to their relatively small proportion in the library A. Compared to the library A, the read population of pCC5.2 significantly increased in library B, and 99.8% of the reads were occupied by pCC5.2 in library C (Supplementary Figure S1A; Supplementary Table S2). We mapped the sequence reads to pCC5.2 to identify the region required for replication, and observed that the region containing ORF B was concentrated in the sequence reads from libraries B and C (Supplementary Figure S1B). ORF B and the flanking region of pCC5.2 (3,242 nt) have been reported to function as replicons to support plasmid replication in *S*. 6803 (Xu and McFadden, 1997; Jin et al., 2018). Our study suggests that this region functions as a replicon that can support plasmid replication in the heterologous cyanobacterium, *S*. 7942.

### 3.2. Phylogenetic analysis of ORF B in pCC5.2 (CyRepA2)

To characterize ORF B in pCC5.2, we performed a sequence-based analysis of the conserved regions and structure of ORF B. Analysis of the SMART and Pfam databases revealed that ORF B has a DUF3854 domain in its N-terminal region. BLAST results revealed that DUF3854-containing proteins are widely conserved among cyanobacteria, not only *Synechococcales,* which contain *S.* 6803 and *S.* 7942, but also *Oscillatoriophycideae*, *Nostocaceae*, *Pleurocapsales*, *Pseudanabaenales*, and *Chroococcidiopsidales* (Figure 1A). The DUF3854-containing proteins formed a distinctly different group from pUH/pANS Rep and RSF1010 RepC and were classified into two main groups. The larger group contained Slr7037 and SYNPCC7002_B0001 proteins encoded in pSYSA (*S*. 6803) and pAQ1 (*S*. 7002), which function as RepA homologs in each organism (Miyasaka et al., 1998; Kaltenbrunner et al., 2022). ORF B belongs to a smaller group, containing proteins in *Microcystis*, *Crocosphaera*, and *Synechocystis*. Although Slr7037 and ORF B are classified into different clades, the predicted structures of these proteins are very similar, with an N-terminal DUF3854 domain and a C-terminal DEXDc domain connected by alpha-helices (Figure 1B). DEXDc domains have been found in proteins with DNA helicase activity, and the function of the DUF3854 domain has been suggested to be related to the Toprim domain conserved in DNA primases (Honda et al., 2006; Jin et al., 2018), although its function remains unknown. Since the group containing Slr7037 is more common in cyanobacteria than the group containing ORF B, we designated Slr7037 as Cyanobacterial Rep protein A (CyRepA1), named ORF B as CyRepA2, and conducted further analysis.

### 3.3. Construction of autonomously replicating plasmid pYS1 in *Synechococcus elongatus* PCC 7942

To evaluate the replication activity of CyRepA2 in *S*. 7942, the expression vector pYS1C-GFP (Supplementary Figure S2) was constructed based on a plasmid containing the minimal region responsible for autonomous replication (1,174–4,683 nt in pCC5.2), ColE1 (replicon for *E. coli*), and the *cat* gene. pYS1C-GFP possesses *gfp* gene under the control of the *trc* promoter and the *lacI* gene, which functions in *S*. 7942, and thus can express GFP in an IPTG-dependent manner. Plasmid pYS1C-GFP was introduced into *S*. 7942 cell and the transformants were used for following analysis. After 48 h of pre-incubation, the cells were cultivated for additional 24 h in presence or absence of 1 mM IPTG and used for analysis. Fluorescence microscopy revealed IPTG-dependent GFP fluorescence in *S*. 7942 cells carrying pYS1C-GFP (Figure 2A). To confirm the structure of pYS1C-GFP in *S*. 7942 cells, DNA was prepared from cells harboring pYS1C-GFP and introduced into *E. coli*. Plasmids were extracted from the resulting *E. coli* transformants and compared with the initial pYS1C-GFP plasmid using restriction enzyme digestion (Figure 3). The results showed that both plasmids were identical, suggesting that pYS1C-GFP was maintained as a plasmid in *S*. 7942 cells.

**Figure 4:**
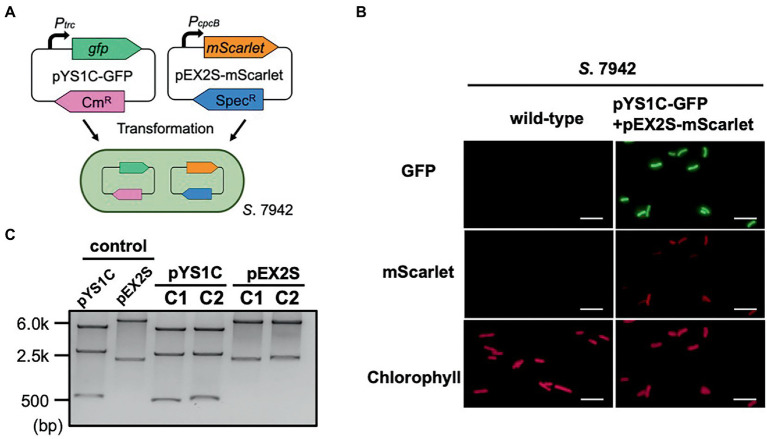
Compatibility of pYS and endogenous plasmid-derived vectors in *S*. 7942. **(A)** Compatibility test in *S*. 7942. pEX2S-mScarlet containing spectinomycin resistance gene (*Sp^R^*) with pHU24/pANS Rep was introduced into *S*. 7942 cell carrying pYS1C-GFP and subjected to microscopy and DNA extraction. **(B)** Fluorescence microscopy. GFP expression was induced in pYS1C-GFP by the addition of 1 mM IPTG (final concentration) to the culture. Images of GFP, mScarlet, and chlorophyll fluorescence are shown. White bar: 10 μm. **(C)** Structure of the plasmids before and after the transformation of *S*. 7942. Plasmid DNA was extracted from *S*. 7942 transformant and transformed into *E. coli*. After extraction from *E. coli*, plasmids were digested with restriction enzymes, and compared with the plasmid before the transformation of *S*. 7942 (control). *Nde*I and *Pst*I (for pYS1C-GFP) and *Sal*I and *Nhe*I (for pEX2S-mScarlet) were used for the digestion. The plasmids before transformation were used as controls. The results of two independent clones are shown as C1 and C2.

To compare the utility of pYS1C-GFP with our previous expression system integrated into the chromosomal neutral site, we constructed an integration plasmid pNSG carrying *gfp* gene. The NS-GFP strain, which expresses GFP from a chromosomal neutral site in an IPTG-dependent manner, was obtained by the transformation of *S*. 7942 with pNSG and GFP expression levels were compared by fluorescence microscopy, flow cytometry, and western blot analysis. Microscopy showed that GFP fluorescence of cells carrying pYS1C-GFP was significantly stronger than that of NS-GFP cells (Figure 2A), although some cells without GFP fluorescence were observed. The expression levels of GFP were quantified and compared by flow cytometry and western blotting analysis, showing that its expression in cells carrying pYS1C-GFP was approximately 10-fold higher than that in NS-GFP cells and was solely dependent on IPTG (Figures 2B,C). These results indicated that the expression system using pYS1 harboring CyRepA2 is more suitable for overexpression than that integrated into the chromosomes in *S*. 7942.

The stability of pYS1C-GFP and the expression level of GFP in *S*. 6803 were also tested using the same approaches as in *S*. 7942, because the use of pCC5.2 as an expression vector has been reported in *S*. 6803 cells (Jin et al., 2018). Consistent with a previous study, we confirmed that pYS1C-GFP was maintained autonomously as a plasmid within *S*. 6803 cells (Figure 3). Although a 5-fold increase in GFP expression was observed with IPTG addition in *S*. 6803, a leaky expression was also detected in the absence of IPTG, in contrast to *S*. 7942 (Supplementary Figure S3). In addition, PCR analysis indicated that the *S*. 6803 pYS1C-GFP transformants contained endogenous pCC5.2 as well, suggesting the instability of pYS1C-GFP (Supplementary Figure S4). Using a more tightly regulated promoter for pYS1 in *S*. 6803 would achieve a more precise expression and stable maintenance of this plasmid.

### 3.4. Compatibility of pYS and endogenous plasmid-derived vector in *Synechococcus elongatus* PCC 7942

Phylogenetic analysis revealed that the sequence of CyRepA2 differs from that of the RepA protein encoded in the plasmid pUH24/pANS of *S*. 7942 (Figure 1A). This led us to assume that pYS can be maintained along with the pHU24/pANS-derived vector in *S*. 7942 cells. To investigate the compatibility of Rep proteins, we constructed the plasmid pEX2S-mScarlet expressing the orange fluorescent protein mScarlet with the spectinomycin resistance marker gene, based on the plasmid pUC303, which was derived from pHU24/pANS. As a result of the transformation of *S*. 7942 cells carrying pYS1C-GFP with pEX2S-mScarlet, we obtained colonies that showed resistance to both chloramphenicol and spectinomycin (Figure 4A). Fluorescence microscopy revealed both GFP and mScarlet fluorescence in *S*. 7942 transformants (Figure 4B), indicating that this strain harbors pEX2S-mScarlet with pYS1C-GFP. In addition, the GFP expression levels of cells carrying these two plasmids were comparable to those of cells harboring only pYS1C-GFP (Supplementary Figure S5). To determine the plasmid structure in *S*. 7942 cells, DNA was extracted, introduced into *E. coli* and selected using chloramphenicol or spectinomycin. Plasmid DNA extracted from the *E. coli* transformants was digested with restriction enzymes. The results showed that the two plasmids retained the same structure as before transformation, confirming that they were independently maintained in *S*.7942 cells (Figure 4C).

### 3.5. Development of expression vectors functioning in *Synechococcus* sp. PCC 7002

**Figure 5:**
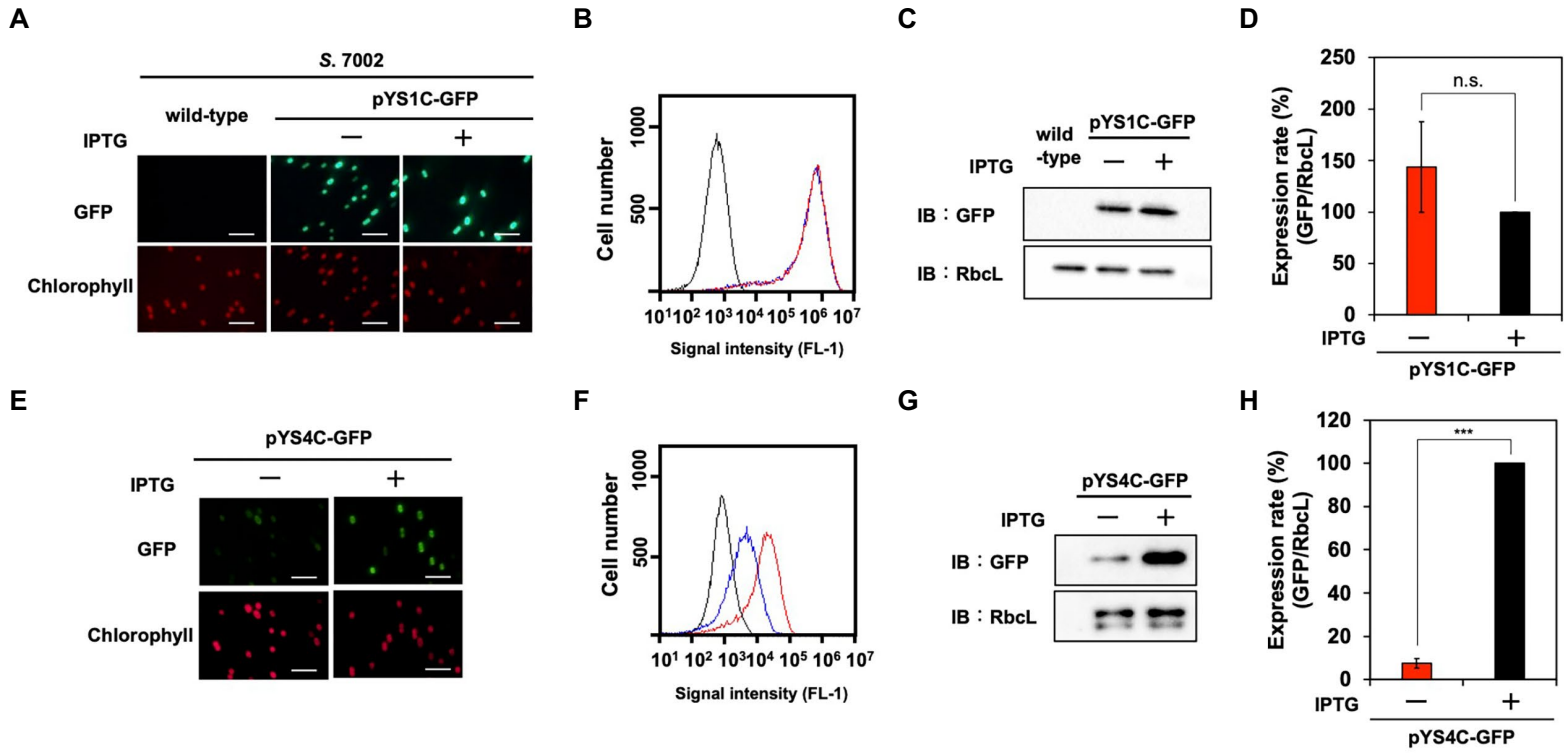
Utilization of expression plasmid pYS1 and pYS4 in *S*. 7002. The GFP expression levels in pYS1C-GFP **(A–D)** and pYS4C-GFP transformants **(E–H)** were analyzed with (+) and without (−) 1 mM IPTG. **(A,E)** Fluorescence microscopy images. The GFP and chlorophyll images are shown. White bar: 10 μm **(B,F)** FACS analysis of GFP fluorescence. Signal intensity of FL-1 indicating GFP fluorescence in *S*. 7002 wild-type (black), pYS- or pYS4-GFP in the presence (red) and absence (blue) of IPTG are shown. **(C,G)** Western blotting analysis. The protein extracts obtained from *S*. 7002 cells were subjected to SDS-PAGE and analyzed by western blotting using antibodies against GFP. RbcL was used as an internal control. **(D,H)** Comparison of GFP signals. The signal intensity of GFP obtained from western blot analysis was normalized to that of RbcL, and the ratio of GFP signal in the presence of IPTG was set to 100. Bars represent the mean ± SEM (*n* = 3; ^***^p < 0.001).

A DUF3854-containing protein was predicted to be encoded in the pAQ1 plasmid of the model marine cyanobacterium *S*. 7002 (SYNPCC7002_B0001), which is closely related to CyRepA1 rather than CyRepA2 (Figure 1A). Thus, we tested the availability of pYS1 in *S*. 7002 using a similar procedure for both *S*. 7942 and *S*. 6803. Similar to the previous two cyanobacteria, significant GFP expression and plasmid maintenance were observed in *S*. 7002 (Figures 3, 5A–D). To test the compatibility between pYS plasmid and endogenous pAQ1 in *S*. 7002 cells, we performed PCR analysis using specific primer sets for the amplification of pYS1C-GFP and pAQ1. The results showed that pAQ1 was stably retained in the pYS1C-GFP transformant (Supplementary Figure S4), indicating that the replication system of the CyRepA2-type can be coexist with that of the CyRepA1-type despite the similarity of their amino-acid sequences. In contrast to the cases of *S*. 7942 and *S*. 6803, there was no difference between the expression (both fluorescence and amount) of GFP proteins in the presence and absence of IPTG in *S*. 7002 (Figures 5A–D), indicating that repression of the *trc* promoter by LacI does not work at all in *S*. 7002, as reported previously (Markley et al., 2015). We noted a broad distribution of the weaker fluorescence signal in the FACS profile of *S*. 7002 (Figure 5B), suggesting that there were differences in the GFP expression levels varies in each cell, which is consistent with the GFP fluorescence observed by microscopy.

To improve the induction system of pYS1C-GFP in *S*. 7002 cells, the *trc* promoter was replaced with the *clac143* promoter, which enables IPTG-dependent regulation in *S*. 7002 (Markley et al., 2015). The resulting plasmid, designated pYS4C-GFP, was introduced into *S*. 7002 and GFP expression was tested in the presence or absence of IPTG. SDS-PAGE analysis demonstrated that the expression level of GFP could be controlled by IPTG (Figures 5E–H). Comparison of the GFP fluorescence intensity of pYS1C-GFP and pYS4C-GFP strains in the presence of IPTG by flow cytometry showed that the fluorescence of the cells carrying pYS1C-GFP was more than one order of magnitude higher than that of cells carrying pYS4C-GFP, indicating that pYS1 can be used as an overexpression system and pYS4 can be used in the situation when strict control of expression is required (Figures 5B,F).

### 3.6. Utilization of pYS vector In *Anabaena* sp. PCC 7120

**Figure 6:**
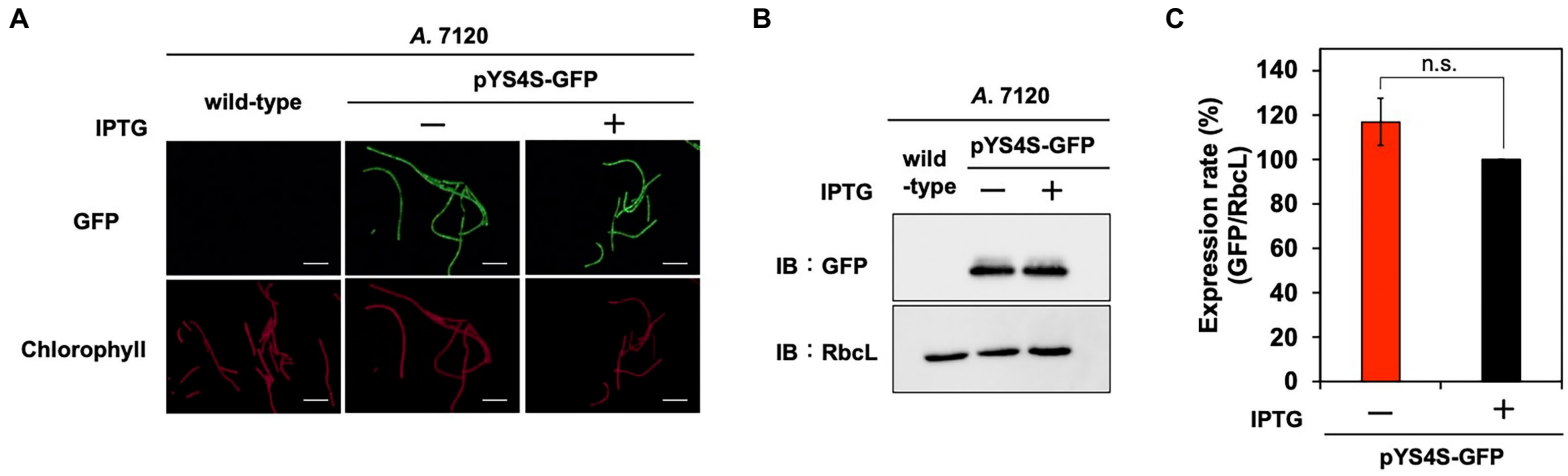
Utilization of pYS in *A*. 7120. GFP expression levels in pYS4S-GFP transconjugants were analyzed with (+) and without (−) 1 mM IPTG. **(A)** Fluorescence microscopy images. The GFP and chlorophyll images are shown. White bar: 20 μm. **(B)** Western blotting analysis. The protein extracts obtained from *A*. 7120 cells were subjected to SDS-PAGE and analyzed by western blotting using antibodies against GFP and control RbcL. **(C)** Comparison of GFP signals. The GFP signals obtained from western blotting analysis were normalized to those of the RbcL signal, and the ratio of the GFP signal in the presence of IPTG was set to 100. Bars represent the mean ± SEM (*n* = 3).

To further test the utility of the pYS plasmid, we introduced it *via* conjugation to filamentous nitrogen-fixing cyanobacterium *A*. 7120, which belongs to *Nostocaceae,* a phylogenetic group distinct from *Synechococcales* including *S*. 6803, *S*. 7942, and *S*. 7002. For the conjugative transport of the pYS plasmid to *A*. 7120, which requires a helper plasmid pRL carrying the *cat* marker gene, we replaced the *cat* gene in pYS1C- and pYS4C-GFP with the spectinomycin resistance marker gene and named the resulting plasmids pYS1S-GFP and pYS4S-GFP, respectively. We successfully isolated 10 clones of *A*. 7120 transconjugants carrying pYS4S-GFP, suggesting that this plasmid could be transported to *A*. 7120 cells by conjugation at a low frequency. On the other hand, a transconjugant carrying pYS1S-GFP was not obtained. This may be due to the overexpression of GFP by the *trc* promoter, as in *S*. 7002, which could be too strong and cytotoxic in *A*. 7120 cells. In the pYS4S-GFP transconjugants, GFP fluorescence was observed in every filamentous cell and the structure of pYS4S-GFP was identical to that of the plasmid before transconjugation (Figures 3, 6A), suggesting that the pYS4-derived vector was maintained stably in *A*. 7120 cells; however, in contrast to *S*. 7002, there was no difference between GFP expression in the presence and absence of IPTG (Figures 6B,C).

## 4. Discussion

Cyanobacteria, which grow by absorbing CO_2_ through photosynthesis, are promising hosts for carbon-neutral material production. However, even with model cyanobacteria, such as *S*. 7942 and *S*. 6803, only a limited number of vectors are available for genetic engineering. Library screening of autonomously replicating regions revealed that the CyRepA2-containing region derived from *S*. 6803 functions in *S*. 7942 and can be used in overexpression vector that function in a wide range of cyanobacterial species.

Our AR-seq analysis using the *S*. 6803 genomic library revealed that the open reading frame (ORF) of CyRepA2 and its upstream region are required for autonomous replication in *S*. 7942 (Supplementary Figure S1B; Supplementary Table S3). This is the first report showing that the CyRepA2 region has autonomous replication activity in heterologous cyanobacteria as well as in its original host, *S*. 6803 (Jin et al., 2018). Although only one autonomous replication region was obtained from this screening, it is possible to identify new Rep and autonomously replicating regions by changing the genomic library and host organisms used for AR-seq. Given its versatility, AR-seq could be used to identify autonomously replicating initiation regions not only in cyanobacteria but also in a variety of other organisms.

It has been reported that pCC5.2 exhibits rolling-circle replication (RCR; Xu and McFadden, 1997). Many RCR plasmids have been shown to replicate in species, genera, or even phyla other than those from which they were isolated. The simplicity of RCR initiation, with only the plasmid-encoded Rep protein participating in origin recognition and priming of leading strand synthesis, is thought to underlie the unusual promiscuity of plasmids (del Solar et al., 2014). Consistent with these observations, the pYS plasmid constructed in this study based on pCC5.2 also exhibited a wide host range, similar to the RCR-employing pSOMA plasmids derived from pCA2.4 and pCB2.4 (Opel et al., 2022). RCR plasmids appear to be suitable for expression because of their small size, high copy number, and the few specific factors involved in replication. Especially in *S*. 7942, GFP expression was 10-fold higher than that in chromosome-based expression systems, which can be tightly controlled by IPTG. These results strongly suggest that the pYS vectors constructed in this study are exceptional genetic engineering tools for potential use at an industrial level in cyanobacteria.

Other applications of RCR plasmids include their use in combination with other plasmids. In this study, we showed that pYS derived from pCC5.2 can be harbored simultaneously with pEX2, derived from an endogenous plasmid in *S*. 7942. Furthermore, we demonstrated that pAQ1 can be maintained along with pYS1C-GFP in *S*. 7002 cells (Supplementary Figure S4), indicating that pYS can be used together with pAQ-based expression systems. It is known that pCC5.2-derived vector pSCB can coexist with the RSF1010-based vector (Jin et al., 2018), and pSOMA10 derived from pCA2.4 can be harbored with pSOMA16 (derived pCB2.4) or RSF1010-based vectors in *S*. 6803 cells (Opel et al., 2022). The utilization of plasmids in various combinations would allow for complex genetic modifications in cyanobacteria, such as metabolic engineering. Further research on these RCR plasmids will enable the development of more suitable plasmids for material production in cyanobacteria.

Even though pYS4S-GFP does not contain the typical origin of transfer, we have succeeded to obtain a transconjugant of *A*. 7120 that possesses this plasmid and shows GFP fluorescence (Figures 3, 6A). The pYS plasmid has inverted repeat sequences, including the terminator, *lacO* operator, and ColE1, which were assumed to act as the transfer origin of conjugation. The mechanism of conjugation of this plasmid should be clarified in the future. It would be possible to increase the efficiency of conjugation by adding a typical origin of transfer.

Structural prediction indicated that CyRepA is a multi-domain protein (Figure 1B). Interestingly, considering the evolution of the CyRepA protein, CyRepA2 has a DUF3854 domain related to the Toprim domain observed in DNA primase, even though RCR does not require synthesis of a primer RNA. The predicted structure of CyRepA2 is very similar to that of CyRepA1 (Figure 1B). However, the two CyRepAs were compatible with *S*. 6803 cells because pSYSA (coding CyRepA1) and pCC5.2 or pYS1C-GFP (coding CyRepA2) coexist in *S*. 6803 cells (Supplementary Figure S4). In *S*. 7002, the pYS1C-GFP transformant stably retained the pAQ1 plasmid carrying the CyRepA1 homolog, SYNPCC7002_B0001 (Supplementary Figure S4). These observations indicate that CyRepA1 and CyRepA2 function independently as replication factors in cyanobacterial cells. While the size of pSYSA is about 20 times larger than that of pCC5.2, the copy number of pSYSA in *S*. 6803 cells was clearly lower than that of pCC5.2, suggesting that CyRepA1 and CyRepA2 have different properties as replication factors (Nagy et al., 2021). The predicted structures provided important insights into the functional differences between CyRepA1 and CyRepA2. In particular, a clear structural difference between the two was observed in the N-terminal region (Figure 1B). Since CyRepA2 is found in some closely related cyanobacteria, such as *Synechocystis*, *Microcystis*, and *Crocosphaera* (Figure 1A), it may have originated from CyRepA1 in the common ancestor of these species. Future comparisons of CyRepA1 and CyRepA2 will reveal functional differences such as host range and replication activity in cyanobacteria.

## Data availability statement

The data presented in the study are deposited in the DDBJ repository, accession number PRJDB11466.

## Author contributions

SW: design of this study. YS: data curation, experiment, and writing—original draft preparation. YS, KM, KN-M, TC, and SW: methodology, writing, review, and editing. TC and SW: conceptualization, methodology, formal analysis, supervision, and writing, review, and editing. All authors contributed to the article and approved the submitted version.

## Funding

This work was supported by the Ministry of Education, Culture, Sports, Science and Technology of Japan to SW (20K05793) and YS (JP22J13447) and the Advanced Low Carbon Technology Research and Development Program (ALCA) of the Japan Science and Technology Agency (JST; to SW). AR-seq was supported by a Cooperative Research Grant of the Genome Research for BioResource, NODAI Genome Research Center, Tokyo University of Agriculture.

## Conflict of interest

The authors declare that the research was conducted in the absence of any commercial or financial relationships that could be construed as a potential conflict of interest.

## Publisher’s note

All claims expressed in this article are solely those of the authors and do not necessarily represent those of their affiliated organizations, or those of the publisher, the editors and the reviewers. Any product that may be evaluated in this article, or claim that may be made by its manufacturer, is not guaranteed or endorsed by the publisher.
