# Supplementary material for: Characterization of a cyanobacterial rep protein with broad-host range and its utilization for expression vectors
Source: Front Microbiol. 2023 Mar 23;14:1111979. doi: 10.3389/fmicb.2023.1111979 (PMC10079941; doi:10.3389/fmicb.2023.1111979)
Supplement: Supplementary file 1 [file Data_Sheet_1.docx]

Supplementary Material

Characterization of a cyanobacterial Rep protein with broad-host range and its utilization for expression vectors

Yutaka Sakamaki, Kaisei Maeda, Kaori Nimura-Matsune, Taku Chibazakura and Satoru Watanabe*

*** Correspondence:** Corresponding Author: [s3watana@nodai.ac.jp](mailto:s3watana@nodai.ac.jp)

# Supplementary Data

**Supplementary Data 1, Predicted 3D structure data of CyRepA1**

**Supplementary Data 2, Predicted 3D structure data of CyRepA2**

**Supplementary Data 3, Complete nucleotide sequence of pYS1C-GFP.**

**Supplementary Data 4, Complete nucleotide sequence of pYS4C-GFP.**

# Supplementary Figures and Tables

## Supplementary Figures

**Supplementary Figure S1. Screening of** **autonomous** **replication region in *S*. 7942 cell.**

(A) The composition of sequence reads of the *S*. 6803 genomic libraries obtained from AR-seq analysis. Three libraries were sequenced by MiSeq and the reads were mapped to the *S*. 6803 genome (chromosome and 7 plasmids, pSYSM, pSYSX, pSYSA, pSYSG, pCA2.4, pCB2.4, and pCC5.2). The sequence reads were analyzed by the CLC Genomics Workbench ver. 20.0.1. Library A: *S*. 6803 genomic library before screening, Library B: DNA extracted from *S*. 7942 cells transformed with Library A, Library C: DNA extracted from *E. coli* cells transformed with Library B. (B) Mapping results of sequence reads to pCC5.2 as references. Reads that have been successfully mapped to pCC5.2 as a pair are shown as blue or light blue, while if only one of the reads in the pair has been mapped, it is shown as red or green. The reads coverage shows the maximum depth of the mapping reads.


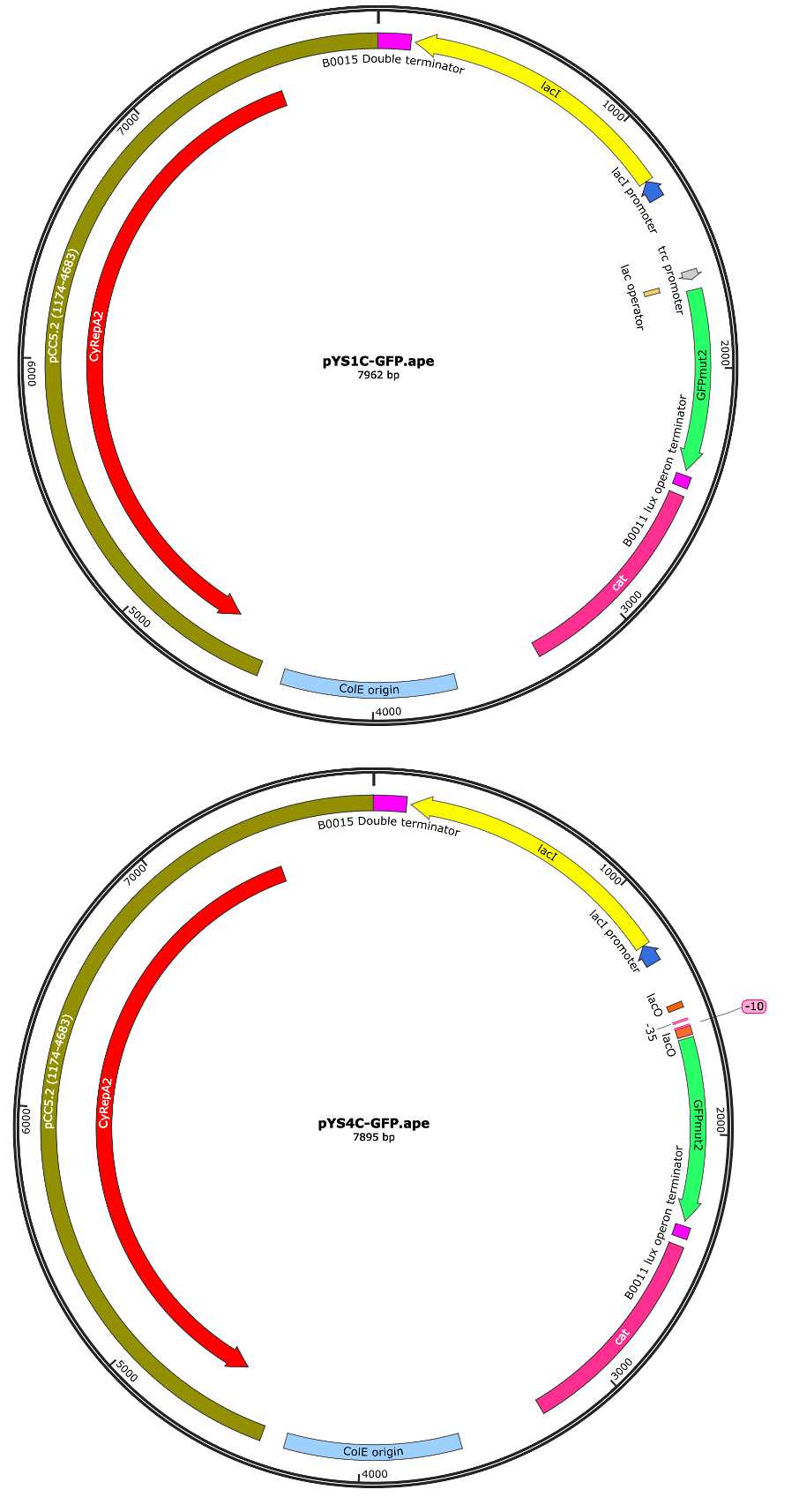


**Supplementary Figure S2. The vector map of pYS1C-GFP and pYS4C-GFP.**

The expression vectors pYS1C-GFP and pYS4C-GFP were constructed from plasmids containing the minimal region of autonomous replication activity obtained by screening (1174-4683 nt including the *CyRepA2* gene of pCC5.2), the *cat* gene, and the *ColE* region. To monitor the expression level in an IPTG-dependent manner, the GFP gene was placed under the *trc* promoter (pYS1) or the *cLac143* promoter (pYS4) containing the *lacO* operator, together with the repressor *lacI* gene. The image of the vector map was drawn using SnapGene software. The vector sequence can be obtained from the Supplementary Data 3 and 4.

**Supplementary Figure S3. Utilization of pYS1 in *S*. 6803.**

GFP expression levels in pYS1C-GFP were analyzed with (+) and without (–) 1 mM IPTG. (A) Fluorescence microscopy images. The GFP and chlorophyll images are shown. White bar:10 μm (B) FACS analysis of GFP fluorescence. Signal intensity of FL1 indicating GFP fluorescence in *S*. 6803 wild-type (black) and pYS1C-GFP transformants in the presence (red) and absence (blue) of IPTG are shown. (C) Western blot analysis. The protein extracts obtained from *S*. 6803 cells were subjected to SDS-PAGE and analyzed by western blotting using antibodies against GFP. (D) Comparison of the GFP signal. The signal intensity of GFP obtained from western blot analysis was normalized to that of RbcL, and the ratio of GFP signal in the presence of IPTG was set to 100. Bars represent the mean ± SEM (*n* = 3) (^*^p < 0.05).


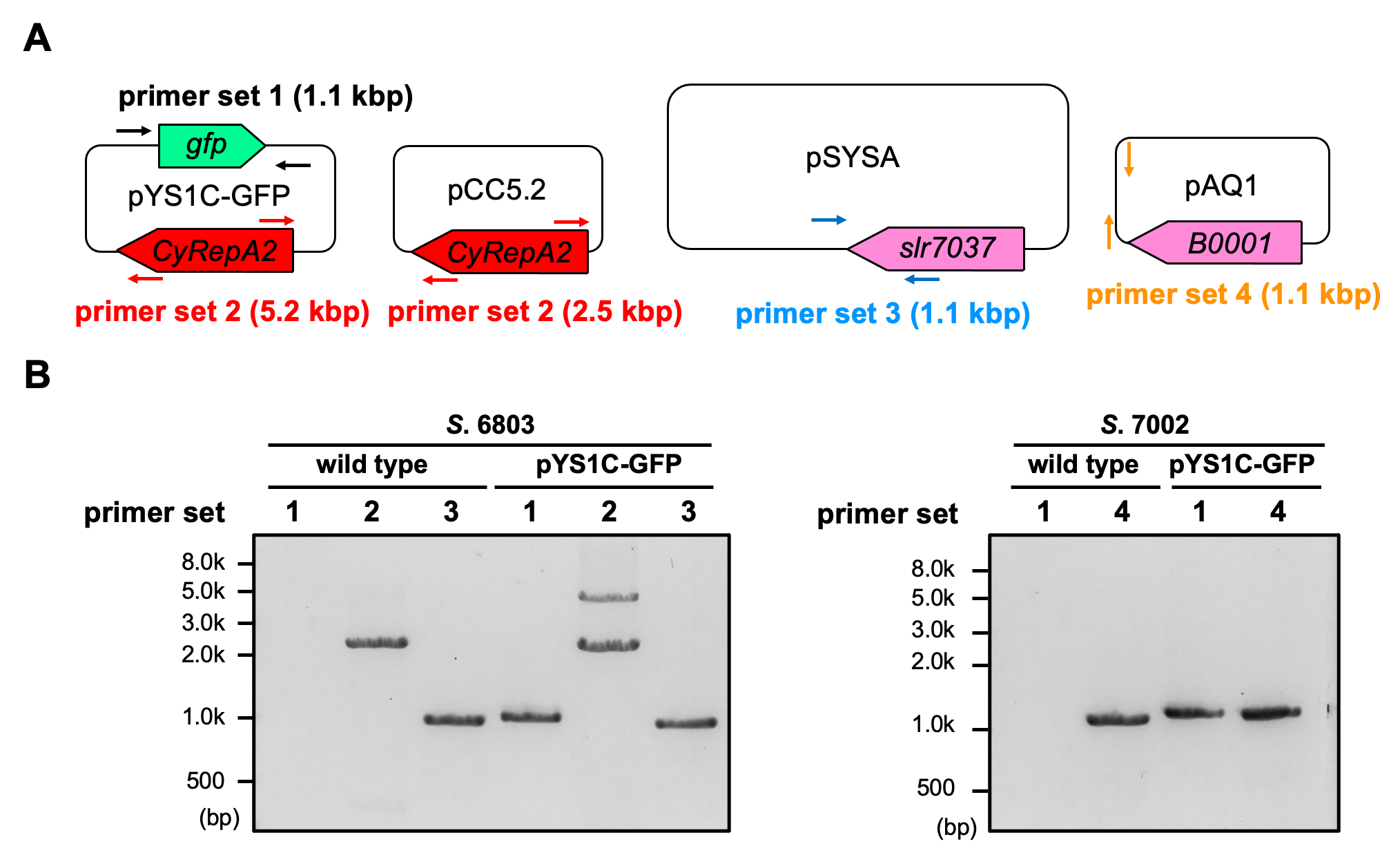


**Supplementary Figure S4.　Compatibility of pYS and endogenous plasmids in *S*. 6803 and *S*.7002.**

(A) A schematic diagram of the analyzed plasmids and the primers used for the PCR analysis. (B) Electrophoresis of PCR products. DNA containing plasmids were extracted from wild type and pYS1C-GFP transformants in *S*. 6803 and *S*.7002 and were PCR-amplified with the appropriate primer sets. pYS1C-GFP: primersF29 and R 30, pCC5.2: primers F 31 and R32, pSYSA: primers F33 and R34, pAQ1: primers F35 and R36 (Supplementary Table S1).

**Supplementary Figure S5. Effect of maintenance of the secondary plasmid on GFP expression levels in pYS plasmid.**

The GFP fluorescence (FL1) was compared. FACS profiles of *S*. 7942 cells harboring pYS1C-GFP (black) and pEX2S-mScarlet in addition to pYS1C-GFP (red) were overlayed. GFP expression was induced by the addition of 1 mM IPTG (final concentration).

**2.2 Supplementary Tables**

**Supplementary Table S1. Oligonucleotide primers used in this study.**

Primer Sequence (5′ to 3′)*^a^*

*Construction of S. 6803 genomic library*

F1 CAACATACGAGCCGGAAGCATAAAGT

R2 GCGGATCCCTGTCAGACCAAGTTTACTCATATATACTTTAGATTGA

F3 GCGGATCCAGAATAAATAAATCCTGGTGTCCCTGTTGA

R4 CCGGCTCGTATGTTGTTACGCCCCGCCCTGCCA

*Plasmid and strain construction*

F5 AGAATAAATAAATCCTGGTGTCCCTGTTG

R6 GATCGTCGTCGCTCAAAAAGAGACTAATAAC

F7 TGAGCGACGACGATCCCAGGCATCAAATAAAACGA

R8 AGCGTCGAGATCCCGGACACCATCGAATGGCGC

F9 CGGGATCTCGACGCTCTCCCTTATGCGACT

R10 GATAACCTCCTAAATTGTTATCCGCTCACAATT

F11 ATTTAGGAGGTTATCATGGAATTCAGTAAAGGAGAAGAACTTTTC

R12 GTATTACTAGTAAGCTTATTTGTATAGTTCATCCATGCCATGTG

F13 GCTTACTAGTAATACTGCAGAGAGA

R14 GGATTTATTTATTCTCACATTTCCCCGAAAAGTG

F15 TGTAATTGACATAAGTCCCATCACCGTTGTATAAATGTGTGGAATTGTGAGCGGATAACAATTTCACACAATGGAATTCAGTAAAGGAGAAGAACTTTTCACTG

R16 TGGGACTTATGTCAATTACATCTTGTTAATTTTATTCCTGCTTTTTTGTTAAGAATTCCGAATTGTGAGCGCTCACAATTCGGGCTCATGAGCGCTTGTTTC

F17 CAACATACGAGCCGGAAGCATAAAGTGTAA

R18 CACATTTCCCCGAAAAGTGCCACCTGA

F19 TTTCGGGGAAATGTGCGCAGCGGTGGTAACGGCG

R20 TGCTTCCGGCTCGTATGTTGTTATTTGCCGACTACCTTGG

F21 GGCGTCGACAGTAAAGGAGAAGAACTTT

R22 GGCAAGCTTTTATTTGTATAGTTCATCC

F23 AACACCTTCGGGAGAGCCTGTTAACACT

R24 CATAGTCGAGTTACGGATCTGCAAGTCAACAGCCGCG

F25 AACTCTTAGATCTGCCACCGCCGGACATCAGCGCTAG

R26 TCTCCCGAAGGTGTTTCAAACATGAGAATTACAACTTATATCG

F27 CGTAACTCGACTATGCTTGTAAACCGT

R28 GCAGATCTAAGAGTTTGTAGAAACGCAAAAAGGCC

*Confirmation of the plasmid structure*

F29 CCGGCTCGTATAATGTGTGG

R30 GAGCAACTGACTGAAATGCCTCA

F31 ATTGTCTGTAGGTAAGTTTTTTAGCGTC

R32 TGAGAAGACTATCCTGCCCAAC

F33 TTGCTCCCGCCACATGGT

R34 GCCATTCTTTTCCTCCATCACTGCGGTGG

F35 TGATCGAAATACTCGTTGTGCAG

R36 GAAACAGAAAATCTAAAGACCAACCCG

**Supplementary Table S2. Sequencing results of *S*. 6803 genomic libraries.**

|  | Library A | Library B | Library C |
| --- | --- | --- | --- |
| chromosome (3,570 kbp) | 80531 (61.851%) | 3734 (5.853%) | 1579 (0.161%) |
| pSYSM (120 kbp) | 4722 (3.627%) | 52 (0.082%) | 18 (0.002%) |
| pSYSX (106 kbp) | 11471 (8.810%) | 119 (0.187%) | 128 (0.013%) |
| pSYSA (103 kbp) | 4666 (3.584%) | 24 (0.038%) | 12 (0.001%) |
| pSYSG (44.3 kbp) | 960 (0.737%) | 20 (0.031%) | 14 (0.001%) |
| pCA2.4 (2.4 kbp) | 1265 (0.972%) | 12 (0.019%) | 16 (0.002%) |
| pCB2.4 (2.4 kbp) | 517 (0.397%) | 9 (0.014%) | 9 (0.001%) |
| **pCC5.2 (5.2 kbp)** | **26069 (20.022%)** | **59821 (93.777%)** | **981084 (99.819%)** |
| Total | 130201 (100%) | 63791 (100%) | 982860 (100%) |

Sequencing reads in each library were mapped to the *S*. 6803 genome (chromosome and 7 plasmids, pSYSM, pSYSX, pSYSA, pSYSG, pCA2.4, pCB2.4, and pCC5.2). The number of reads and ration in each library were shown.

**Supplementary Table S3. The region contained in the transformants of library B.**

| Clone number | Name | Genbank ID | Strat point | End point | Length (bp) |
| --- | --- | --- | --- | --- | --- |
| 1 | pCC5.2 | CP003272.1 | 1174 | 4726 | 3553 |
| 2 | pCC5.2 | CP003272.1 | 1174 | 5133 | 3960 |
| 3* | pCC5.2 | CP003272.1 | 1174 | 4683 | 3510 |
| 4 | pCC5.2 | CP003272.1 | 4763 | 1174 | 3590 |
| 5 | pCC5.2 | CP003272.1 | 1174 | 4763 | 3590 |
| 6 | pCC5.2 | CP003272.1 | 895 | 5110 | 4216 |
| 7 | pCC5.2 | CP003272.1 | 895 | 4674 | 3780 |
| 8 | pCC5.2 | CP003272.1 | 1174 | 5159 | 3986 |
| 9 | pCC5.2 | CP003272.1 | 895 | 5087 | 4193 |
| 10 | pCC5.2 | CP003272.1 | 1174 | 5159 | 3986 |
| 11 | pCC5.2 | CP003272.1 | 5159 | 1174 | 3986 |
| 12 | pCC5.2 | CP003272.1 | 1174 | 4763 | 3590 |
| 13 | pCC5.2 | CP003272.1 | 895 | 5159 | 4265 |
| 14 | pCC5.2 | CP003272.1 | 1177 | 1202 | 5188 |

Fourteen of *E. coli* colonies were selected from transformants of Library B and the sequences of their insert regions were determined by Sanger sequencing. All sequenced regions were matched to the plasmid pCC5.2 in *S*. 6803. The base numbers in the pCC5.2 sequence (Genbank ID: CP003272.1) at the start point and end point of the insert regions were shown along with the length of the inserts.

*Clone 3, which contains minimum insert region, was used for constructing pYS vectors.
