# Supplementary material for: Characterization of a cyanobacterial rep protein with broad-host range and its utilization for expression vectors
Source: Front Microbiol. 2023 Mar 23;14:1111979. doi: 10.3389/fmicb.2023.1111979 (PMC10079941; doi:10.3389/fmicb.2023.1111979)
Supplement: Supplementary file 2 [file Data_Sheet_2.zip › Supplementary Data/Supplementary Data 3_pYS1C-GFP.docx]

**Supplementary Data 3, Complete nucleotide sequence of pYS1C-GFP.** *trc* promoter (red), lacO (gray), *gfp*^mut2^ (green), *cat* gene (pink), *ColE* (light blue), pCC5.2 region (yellow)

CCAGGCATCAAATAAAACGAAAGGCTCAGTCGAAAGACTGGGCCTTTCGTTTTATCTGTTGTTTGTCGGTGAACGCTCTCTACTAGAGTCACACTGGCTCACCTTCGGGTGGGCCTTTCTGCGTTTATACATTAATTGCGTTGCGCTCACTGCCCGCTTTCCAGTCGGGAAACCTGTCGTGCCAGCTGCATTAATGAATCGGCCAACGCGCGGGGAGAGGCGGTTTGCGTATTGGGCGCCAGGGTGGTTTTTCTTTTCACCAGTGAGACGGGCAACAGCTGATTGCCCTTCACCGCCTGGCCCTGAGAGAGTTGCAGCAAGCGGTCCACGCTGGTTTGCCCCAGCAGGCGAAAATCCTGTTTGATGGTGGTTAACGGCGGGATATAACATGAGCTGTCTTCGGTATCGTCGTATCCCACTACCGAGATATCCGCACCAACGCGCAGCCCGGACTCGGTAATGGCGCGCATTGCGCCCAGCGCCATCTGATCGTTGGCAACCAGCATCGCAGTGGGAACGATGCCCTCATTCAGCATTTGCATGGTTTGTTGAAAACCGGACATGGCACTCCAGTCGCCTTCCCGTTCCGCTATCGGCTGAATTTGATTGCGAGTGAGATATTTATGCCAGCCAGCCAGACGCAGACGCGCCGAGACAGAACTTAATGGGCCCGCTAACAGCGCGATTTGCTGGTGACCCAATGCGACCAGATGCTCCACGCCCAGTCGCGTACCGTCTTCATGGGAGAAAATAATACTGTTGATGGGTGTCTGGTCAGAGACATCAAGAAATAACGCCGGAACATTAGTGCAGGCAGCTTCCACAGCAATGGCATCCTGGTCATCCAGCGGATAGTTAATGATCAGCCCACTGACGCGTTGCGCGAGAAGATTGTGCACCGCCGCTTTACAGGCTTCGACGCCGCTTCGTTCTACCATCGACACCACCACGCTGGCACCCAGTTGATCGGCGCGAGATTTAATCGCCGCGACAATTTGCGACGGCGCGTGCAGGGCCAGACTGGAGGTGGCAACGCCAATCAGCAACGACTGTTTGCCCGCCAGTTGTTGTGCCACGCGGTTGGGAATGTAATTCAGCTCCGCCATCGCCGCTTCCACTTTTTCCCGCGTTTTCGCAGAAACGTGGCTGGCCTGGTTCACCACGCGGGAAACGGTCTGATAAGAGACACCGGCATACTCTGCGACATCGTATAACGTTACTGGTTTCACATTCACCACCCTGAATTGACTCTCTTCCGGGCGCTATCATGCCATACCGCGAAAGGTTTTGCGCCATTCGATGGTGTCCGGGATCTCGACGCTCTCCCTTATGCGACTCCTGCATTAGGAAGCAGCCCAGTAGTAGGTTGAGGCCGTTGAGCACCGCCGCCGCAAGGAATGGTGCATGCAAGGAGATGGCGCCCAACAGTCCCCCGGCCACGGGGCCTGCCACCATACCCACGCCGAAACAAGCGCTCATGAGCCCGAAGTGGCGAGCCCGATCTTCCCCATCGGTGATGTCGGCGATATAGGCGCCAGCAACCGCACCTGTGGCGCCGGTGATGCCGGCCACGATGCGTCCGGCGTAGAGGATCGAGATCTCGGGTACCGAGCTGTTGACAATTAATCATCCGGCTCGTATAATGTGTGGAATTGTGAGCGGATAACAATTTAGGAGGTTATCATGGAATTCAGTAAAGGAGAAGAACTTTTCACTGGAGTTGTCCCAATTCTTGTTGAATTAGATGGTGATGTTAATGGGCACAAATTTTCTGTCAGTGGAGAGGGTGAAGGTGATGCAACATACGGAAAACTTACCCTTAAATTTATTTGCACTACTGGAAAACTACCTGTTCCATGGCCAACACTTGTCACTACTTTCGCGTATGGTCTTCAATGCTTTGCGAGATACCCAGATCATATGAAACAGCATGACTTTTTCAAGAGTGCCATGCCCGAAGGTTATGTACAGGAAAGAACTATATTTTTCAAAGATGACGGGAACTACAAGACACGTGCTGAAGTCAAGTTTGAAGGTGATACCCTTGTTAATAGAATCGAGTTAAAAGGTATTGATTTTAAAGAAGATGGAAACATTCTTGGACACAAATTGGAATACAACTATAACTCACACAATGTATACATCATGGCAGACAAACAAAAGAATGGAATCAAAGTTAACTTCAAAATTAGACACAACATTGAAGATGGAAGCGTTCAACTAGCAGACCATTATCAACAAAATACTCCAATTGGCGATGGCCCTGTCCTTTTACCAGACAACCATTACCTGTCCACACAATCTGCCCTTTCGAAAGATCCCAACGAAAAGAGAGACCACATGGTCCTTCTTGAGTTTGTAACAGCTGCTGGGATTACACATGGCATGGATGAACTATACAAATAAGCTTACTAGTAATACTGCAGAGAGAATATAAAAAGCCAGATTATTAATCCGGCTTTTTTATTATTTAGACGTCAGGTGGCACTTTTCGGGGAAATGTGAGAATAAATAAATCCTGGTGTCCCTGTTGATACCGGGAAGCCCTGGGCCAACTTTTGGCGAAAATGAGACGTTGATCGGCACGTAAGAGGTTCCAACTTTCACCATAATGAAATAAGATCACTACCGGGCGTATTTTTTGAGTTATCGAGATTTTCAGGAGCTAAGGAAGCTAAAATGGAGAAAAAAATCACTGGATATACCACCGTTGATATATCCCAATGGCATCGTAAAGAACATTTTGAGGCATTTCAGTCAGTTGCTCAATGTACCTATAACCAGACCGTTCAGCTGGATATTACGGCCTTTTTAAAGACCGTAAAGAAAAATAAGCACAAGTTTTATCCGGCCTTTATTCACATTCTTGCCCGCCTGATGAATGCTCATCCGGAATTCCGTATGGCAATGAAAGACGGTGAGCTGGTGATATGGGATAGTGTTCACCCTTGTTACACCGTTTTCCATGAGCAAACTGAAACGTTTTCATCGCTCTGGAGTGAATACCACGACGATTTCCGGCAGTTTCTACACATATATTCGCAAGATGTGGCGTGTTACGGTGAAAACCTGGCCTATTTCCCTAAAGGGTTTATTGAGAATATGTTTTTCGTCTCAGCCAATCCCTGGGTGAGTTTCACCAGTTTTGATTTAAACGTGGCCAATATGGACAACTTCTTCGCCCCCGTTTTCACCATGGGCAAATATTATACGCAAGGCGACAAGGTGCTGATGCCGCTGGCGATTCAGGTTCATCATGCCGTTTGTGATGGCTTCCATGTCGGCAGAATGCTTAATGAATTACAACAGTACTGCGATGAGTGGCAGGGCGGGGCGTAACAACATACGAGCCGGAAGCATAAAGTGTAAAGCCTGGGGTGCCTAATGAGTGAGCTAACTCACATTAATTGCGTTGCGCTCACTGCCCGCTTTCCAGTCGGGAAACCTGTCGTGCCAGCTGCATTAATGAATCGGCCAACGCGCGGGGAGAGGCGGTTTGCGTATTGGGCGCTCTTCCGCTTCCTCGCTCACTGACTCGCTGCGCTCGGTCGTTCGGCTGCGGCGAGCGGTATCAGCTCACTCAAAGGCGGTAATACGGTTATCCACAGAATCAGGGGATAACGCAGGAAAGAACATGTGAGCAAAAGGCCAGCAAAAGGCCAGGAACCGTAAAAAGGCCGCGTTGCTGGCGTTTTTCCATAGGCTCCGCCCCCCTGACGAGCATCACAAAAATCGACGCTCAAGTCAGAGGTGGCGAAACCCGACAGGACTATAAAGATACCAGGCGTTTCCCCCTGGAAGCTCCCTCGTGCGCTCTCCTGTTCCGACCCTGCCGCTTACCGGATACCTGTCCGCCTTTCTCCCTTCGGGAAGCGTGGCGCTTTCTCAATGCTCACGCTGTAGGTATCTCAGTTCGGTGTAGGTCGTTCGCTCCAAGCTGGGCTGTGTGCACGAACCCCCCGTTCAGCCCGACCGCTGCGCCTTATCCGGTAACTATCGTCTTGAGTCCAACCCGGTAAGACACGACTTATCGCCACTGGCAGCAGCCACTGGTAACAGGATTAGCAGAGCGAGGTATGTAGGCGGTGCTACAGAGTTCTTGAAGTGGTGGCCTAACTACGGCTACACTAGAAGGACAGTATTTGGTATCTGCGCTCTGCTGAAGCCAGTTACCTTCGGAAAAAGAGTTGGTAGCTCTTGATCCGGCAAACAAACCACCGCTGGTAGCGGTGGTTTTTTTGTTTGCAAGCAGCAGATTACGCGCAGAAAAAAAGGATCTCAAGAAGATCCTTTGATCTTTTCTACGGGGTCTGACGCTCAGTGGAACGAAAACTCACGTTAAGGGATTTTGGTCATGAGATTATCAAAAAGGATCTTCACCTAGATCCTTTTAAATTAAAAATGAAGTTTTAAATCAATCTAAAGTATATATGAGTAAACTTGGTCTGACAGTAATGCCCTGCACTTCATCCTTAACTGGTATCGGGACTCTTAGGTGAGTGGTGAGATACGGGGCATGATGTCCCCTTCCCCCTTTGGGTTTATCTATGCCCTGCATGGGTTTAGCCTGTTAACGGGATTATATTCCCATCATAACTCCCTGTTTTTGGTATCCAGTTCCTAGGCTTGATTAATTAATAAGGATTCAGTGGATACGGTATCAGAGTGATACAAAATAGAATCCCGCTCAAACCACCGGGAAAAAATAGCCGATCGCCCATCGGGGTCAATATTGGCGGGGCCATAATGTCGCTTAGTATTGCCCCGTTCCCCAAACCGGCCCAGATACTCAAGATGTAAACCCATTTTTTTCAAGACAGATTGCACGAAGGCGATCGCCCCTTTGTCAGGGTTGATCGTGATGCCCAAGGCCTGCTTAATTTCTGCCTTGTTGGGCAGGATAGTCTTCTCAAACCAATTGGCTAGGGAATCCTTGGAAAATTCCCCTATCCCGGTAAGGAATCTTTCAAAGCCCAATACTTTAAGGAAGTGAACGGGGACGGTGAGAGTCTTCCGGCTAGCATCCTTGGCACAAACCTTTCCATCTTCCCCGGCCAAATTTTGGAGCCTTTGGCTGTCCCTTGCCTGTAAAAATTCTGCACCGGTGGTGAAGTAATAATGCAGGGTCAATTGGGCCAGCCATCCTTGATCACATTTCTCCACATCTTCTGGGGTTATGTCATCGGTAGCCAGGGCACGGCAAAGATCTCCCTTCCGCTCTTGATATCGTTCCGCTTCCGTCTTAACTTGCTTCCGCTTCAAACTTTCATAGGTGAAGTCATCGGGATTAAGGCTCTCGGCGATCGCCGTGGTTTCCTTTCCATAGTTATGGTCACGGATAGCTTTAATGCGATCAGCTACGTCTTCCGCCCCGGTGACCATATCTGGGTCAGGTTCTTTCTGTGTGTACCCTTCTTCCCCTAATTTTTCAAGAGTAGTTGATTTTAAGCTTTTCTTCCCTTGATTGTGCAGAGCCGCCATAATAGCCCAGGTTTTAAGATGCTCTGGCTTCTCATCATCAAACAGGGTTAAATTGTCGGCTGTATTCAATGCCCCGATAGTAGCCTTGGCTTTCTTATTCTCCACATTAAGGATATAGCGAGGGTTAGTTTCCCCGCCAGCAATAAAACAATTATTAGGGGCACGATTGCGAATCCAAACATGGCGATCCACATCGGGACGATACCTTTCTAATCCTTGGCAGAATTGCTCTACTGTTTGTGTTCCCTGTCCGAATCCATAGACTTCATCAAAATAGGGCTTATCAATGCTGATTCCCGTCTCTATTACCGGGGACGTGATGACAATATCCTTGTCTTCTAAATATCTATCTAAGTTATCCATACAGCCATAGGCAGGGTTAGTCTTATCGCTTACTGTATGGGCATCTAAGACCCCTACTGACTTATCAGGGAATAACATTTTGAATAAGTCCCCTAGGTTAGTTGTTGAATAGATAGACTGTGCTTTTTGTGCCCCGGTGCAAATCTGTATTTTTTTACCATTAGCAACAGTATTAATAGCGGAAGTAAGCAAATCTTCCGGGGAATTATAAACAATCAAATTACGCTTATCCTGCACTGGCTTAAACGTGTTAACGACAATATAGCATTCTGCCTTACCACCGATTAAGTCTTGGACATACTTGATAGTCACCGGGGACAAATCAGCATCAGACAAAATAATCTTGCCCCCCGAATCAGCGGCGGCGATCAATATTTCTTGGAAGCGGGCCAAGATGGCTGGTTTATGTTTGCCCAGGTTTCCCCGGGAACTATCTAATAATTCCCATATCAATTGCTCGCATTCATCAAGGATGATGTCATGGCCCCGGAATGTTTCGGGGTCAAGTTTCAGGATGCTATCAATGCACAATCCGATTCCTAAGGCCCCATTAGTGTCGCTCTCGGACAAATCTCTGATGTAGTCCACACCAAACCGGCCGGATAGGGCAATGCCTAGTTGAATTCGGTGAGTGAGTGGATAAGTCCGGCGAAAATCTTCTCGCCGTTGCTCCACCATTTTTGAGATCGCTTCCGTCTTTCCCGTGCCCTTGGCTGATTTAATCCCGATTATTTTGGCGGTGGTAGGGGCATTAATGTTGCTTAGGTATCTTTCGTGCCGGGTTTGATTCACCCATTGGGATAGGTCAGCATACGGCTTGTTTTGGAACTGTTCTAGGCTGATGCCCCGAGCGATCGCCCGGTGAAATTTAGCAGGGTTATTGGCGATCAAGTCATCAATGCCCTTGCCGTCTTCCCCGTTCCATTGGCAGATCTTGACCGTTGCCTTAGCGTGATAGGTCAGGTTACGAGCCAGCCGTTTTGTGCCCTTAAAGACTGCCTTTCGTCCCTTGCTACCCTTTGCATCCTGGTCATAGGCGATCGCTACTTCTCGCCCCTGCACATAGGGCAATAATGACGGTTTGATGGTTAGACCATCGTTGCCACATAAACAGCCATAGAGACTCAGGGCCACGTAACCTTGGCTGATGGCGGCTAAAGCTTTCTTCCCCCCTTCCGTCACAATCAAGGGAATCTCAGGATGATCCACAAACCAGGGCCAAAAATCTTGACCATCTTCCGGTGGTTTGACCCCATGCTTTTCGGCGATCGCCAAAATGATCCGGCGGGGAATTGTTGGCAGATAGGGCACGTCCCCTATGCCTGTCGGTGCTAGATACTGCCCTGACCGCTTGCCCGTCTTGTCTTCACCAAAGATTTTTACCTGCCATACGTTCCCATTCTCAGACCGGAAAATGGCGGCCTGCCTGTCTTCCTTGGCTTGATGTCCAAAGCGGGTAAATTTTCGCCCCAGGGCATCGGCGATCGGCGTTGCCAAGACTTCCTTGGTGATGGGGTCTATCTCAAGATCCGGCACAATCTCCACGTTGGCCCGGAATACGTCAGGGGCGATCGCCGAATCTTCTACAAATTCTGACCGTATTTTTGGGTCATTTTTTGACCCTTGCCATCCGTTGAAATGCTGATGGGCTAAGGCTTGAGAATTTTGGTGGGAAAGACTATAATTGTTCATAGCGGTCTCGGGTTCAAGAGTTGACCGCTTTTCTTTTGGGAATTTTTAGAGCTACAGTAGAGACAATATTTTTGAAGTATTTTGTTGGCTTTTTGGGCCGGGTTATTGTCTGTAGGTAAGTTTTTTAGCGTCAACTAAACTTCTTACGTTTTTTACTGCAGCAATCTTAATTCCCTCCTGAAAAGCTTTTTTTTGTTGGAAATCTTGGACTATTGCCAGAATTGTAACGGATTAGATCGGTCGCTGTCACGCTGTTTTATCGTCATAAAAGTTTATTTAAATTAAATATGTGAGCAATGCTCACGGCTGTCGCTATCCCTTTGTCAAGAGATAATACACTTTGGATTGTCCCCTAAAAGATTGTTTTTATAATCGGCTTATTTTCTCTTTCTGGTGTTATTAGTCTCTTTTTGAGCGACGACGATC
